# Facial feedback on the perception and memory for emotional faces

**DOI:** 10.1007/s00426-026-02307-4

**Published:** 2026-05-11

**Authors:** Ivan Nabil Ras, Monica Bucciarelli, Francesco Ianì, Teresa Limata, Alessia Sutera, Carla Tinti, Susanna Schmidt

**Affiliations:** 1 Department of Psychology, Università degli Studi di Torino, Via Verdi, 10, Turin, 10124 Italy; 2 Centro di Logica, Linguaggio, e Cognizione, Università degli Studi di Torino, Turin, Italy

## Abstract

Our investigation explores the facial feedback hypothesis in relation to the perception and memory of emotional faces. In Experiment 1, participants rated the valence of happy, neutral, and angry faces of different actors while vertically holding a pen either between their teeth (smile-facilitating condition) or between their lips (smile-inhibiting condition). Results indicated that participants in the teeth condition rated happy faces more positively than participants in the lips condition, while no difference was found for angry and neutral faces. Experiment 2 assessed long-term memory for happy, neutral, and angry faces. During encoding, participants saw happy, neutral, and angry faces of different actors. During recognition, while holding a pen either between their teeth or their lips, they had to select the previously seen faces from a set containing the target face (same emotion expression) and two filler faces (other emotion expressions) of the same actor. Results indicated no significant differences both for accuracy and recognition times between the two groups. Taken together, these findings suggest that the pen-in-mouth procedure influences the perception of happy faces only, while the manipulation had no significant effect on memory. Interestingly, angry faces required more time to be evaluated in Experiment 1 and were recognized more accurately in Experiment 2. We discuss these results in relation to the literature on the facial feedback hypothesis and embodied emotion theories.

**Supplementary Information:**

The online version contains supplementary material available at 10.1007/s00426-026-02307-4.

## Facial feedback on the perception and long-term memory for emotional faces

The Facial Feedback Hypothesis (FFH) posits that facial expressions can influence or even trigger emotional experiences, suggesting a crucial bidirectional relationship between facial muscle activity and emotions (Strack et al., 1988). Building on earlier conceptualizations of emotion as bodily processes (e.g., James, 1884), more recent work has explicitly articulated the notion of “embodied emotion” (Prinz, 2004), presenting one of the first modern frameworks to ground emotional experience in bodily responses. Theories of embodied emotion propose that emotional processes are grounded in bodily states, which can shape the perception and recognition of affective stimuli (Price & Harmon-Jones, 2015), and empirical findings suggest that when posture at encoding and retrieval is congruent, recall is facilitated (e.g., Dijkstra et al., 2007). This perspective implies that sensorimotor simulation processes, such as the activation of facial muscles, may directly enhance the perception and recognition of emotional faces (Wood et al., 2016).

Strack et al. (1988) found evidence supporting the FFH in the perception of humorous cartoons, which participants rated more positively when their facial muscle activity was manipulated using the so-called pen-in-mouth procedure to facilitate smiling.

The present investigation uses the pen-in-mouth procedure with two aims. The first aim is to test the FFH in the perception of emotional faces, given their ecological validity and high relevance for social interaction (Wood et al., 2016). Experiment 1 aims to replicates Strack et al.’s (1988) study with different stimuli: happy faces instead of funny cartoons. Moreover, the experiment examines the presence or absence of the facial feedback effect when participants evaluate angry and neutral faces. The second aim is to integrate the FFH with evidence of the congruency effect of bodily states on memory, as suggested by posture congruence studies. Experiment 2 explores the potential beneficial effect on long-term memory when the observer’s facial expression and the observed expression are congruent. In the literature, no studies have tested the FFH for both the perception of emotional faces and their long-term memory within the same methodological framework.

### Facial feedback hypothesis and perception

The FFH has been widely debated in research on the nature of emotions. Although there are several variants of this theory, it is generally based on the idea that muscle feedback from facial expressions plays a causal or at least a modulating role in emotional experience. This idea has several historical roots. Charles Darwin stated, “The free expression by outward signs of an emotion intensifies it. […] Even the simulation of an emotion tends to arouse it in our minds” (1872, p. 366). Based on this premise, several researchers have emphasized the importance not only of facial expression but, more generally, of bodily states in determining subjective emotional experience. One of the most important theories supporting this approach was proposed by James (1884), who argued that emotions are essentially the perception of bodily changes rather than purely mental states: “[…] the more rational statement is that we feel sorry because we cry, angry because we strike, afraid because we tremble, and not that we cry, strike, or tremble, because we are sorry, angry, or fearful […]” (1884, p. 190). According to James, the process of emotional experience begins with an internal or external stimulus that triggers specific changes in the body’s state at behavioral, physiological, and expressive levels. Awareness of these changes gives rise to a subjective emotional experience.

Following Darwin and James, several authors have emphasized the role of bodily states in shaping emotional experience, an idea now commonly subsumed under the concept of “embodied emotion” (Prinz, 2004; see also Price & Harmon-Jones, 2015 for a review). In particular, Tomkins (1962a, b) highlighed the role of facial muscles in generating and modulating emotional experience. His account underscores the critical role of facial feedback in shaping affect, suggesting that an internal or external emotional stimulus triggers an inborn “affect program” (1962a, p. 244) that transmits messages via motor and circulatory pathways to the face; the face then sends sensory feedback to the brain, and when this feedback reaches consciousness, it is experienced as a specific emotion (for a review of more recent developments in affect theory, see Wood et al., 2016).

In line with Tomkins’ idea of innate affect programs, Ekman et al. (1983) hypothesized that emotions consist of coordinated patterns of expressive, physiological, behavioral, and subjective feeling responses, and that activation of one response component can automatically trigger the others. The authors demonstrated this by having participants perform a directed facial action task, in which specific facial muscles were contracted (e.g., for fear: “raise your brows and pull them together”, “now raise your upper eyelids”, “now also stretch your lips horizontally, back toward your ears”, p. 1208). This method was used to demonstrate the existence of an affect program that can be activated by each response component of a specific emotion, producing distinct autonomic nervous system reactions and eliciting the corresponding subjective feelings.

However, despite these findings, Strack et al. (1988) raised methodological concerns. Although Ekman et al. (1983) avoided using emotion labels, their procedure required participants to mimic expressions corresponding to specific emotions, which may have influenced the results. Participants’ awareness of imitating emotional expressions could have induced the corresponding feelings, rather than the facial feedback operating as a purely implicit mechanism. Additionally, this awareness might have prompted compliance with the experimenters’ expectations.

To address these potential shortcomings, Strack et al. (1988) tested the FFH using a novel experimental paradigm. Participants were told that the study examined the difficulty of writing or drawing with a pen held in the mouth, as individuals unable to use their hands might do. One group held the end of a pen vertically with their teeth (facilitating smiling), while the other group held the end of a pen vertically with their lips (inhibiting smiling). This methodological approach was used to ensure that any emotional changes observed were due to the induced facial expressions and not to the participants’ awareness of the true aims of the study. While holding the pen, participants rated the funniness of cartoons. The authors hypothesized that holding the pen between the teeth would facilitate a smile and enhance the feeling of happiness, which in turn would lead to a more positive evaluation of the cartoons. The results confirmed the hypothesis, showing that participants in the teeth condition rated the cartoons as funnier than those in the lips condition. This study is considered revolutionary because of the introduction of the pen-in-mouth procedure: by facilitating or inhibiting a smile without explicit instructions, the authors addressed the criticism of demand characteristics and provided stronger evidence for the FFH.

Nevertheless, various attempts to replicate Strack et al.’s findings have yielded controversial results (for a review, see Coles et al., 2019). To address this inconsistency, Wagenmakers et al. (2016) conducted a registered replication study involving 17 independent research groups, each attempting to replicate Strack et al.’s (1988) original study. The results showed no significant difference between the teeth and lips conditions in participants’ funniness ratings of cartoons. Noah et al. (2018; see also Strack, 2016) hypothesized that the failure to replicate the original study may be due to a critical procedural difference: in the replication study, but not in the original, participants were aware that they were being videotaped. The authors tested this hypothesis by introducing two experimental conditions: one with a camera present and one without. The results supported their hypothesis: the facial feedback effect was significant when no camera was present, but not when a camera was present. These data suggest that awareness of being observed can negatively influence the facial feedback effect.

The findings of Noah et al. (2018) are further supported by Marsh et al. (2019), who conducted a study involving undergraduate students exposed to the pen-in-mouth procedure while evaluating the funniness of cartoons without being video recorded. The authors found a significant difference in humor ratings between the two experimental conditions: participants in the teeth condition rated the cartoons as funnier than those in the lips condition. These results replicated the effect reported by Strack et al. (1988) and provided further evidence in support of the FFH.

Other researchers used similar stimuli to those in our study (emotional facial expressions), but the facial manipulations differed from the original pen-in-mouth procedure. For example, in a study by Blaesi and Wilson (2010), participants viewed 11 photos of the same face on a continuum from smiling to frowning. For each photo, participants were asked to say whether the face was happy or sad. Each participant viewed the stimuli under two conditions. In the Pen condition, they were instructed to hold a pen horizontally (rather than vertically as in Strack et al., 1988) with their teeth, without touching it with their lips. This manipulation was intended to favor a smile. In the No Pen condition, participants received no special instructions. Results showed a lower threshold for perceiving a happy expression in the Pen condition. According to the authors, this demonstrates how simulating a smile lowers the recognition threshold for happy faces (see also Marmolejo-Ramos et al., 2020, Experiment 1).

In another study by Oberman et al. (2007, Experiment 2), participants viewed photos of happy, sad, fearful, and disgusted faces under four conditions: (a) bite (holding a pen horizontally by exerting continuous pressure with the teeth), (b) gum (chewing), (c) lips (holding a pen horizontally with the lips), and (d) rest (baseline). The authors intended the “lips” and “rest” conditions as controls, while the “bite” and “gum” conditions were designed to create irrelevant muscular noise that would interfere with facial mimicry. On each trial, a face appeared on the screen, and participants were asked to rate the expression as conveying happiness, sadness, fear, or disgust. The results showed that the “bite” condition specifically impaired recognition of happy faces and, to some extent, disgusted faces. According to the authors, this can be explained by the mimicry-blocking effect of the “bite” condition. Similar results were reported by Borgomaneri et al. (2020, Experiment 1), who tested participants’ ability to identify happy, fearful, and neutral expressions. They found that biting a pen reduced recognition of happy expressions, but not neutral or fearful ones.

In another study, Ponari et al. (2012, Experiment 1) examined the effects of interfering muscle contractions in the upper or lower half of participants’ faces on their ability to recognize fear, happiness, anger, disgust, sadness, and surprise expressions. They hypothesized that interfering with the face muscles involved in producing a particular expression should impair recognition of that same expression. Participants were assigned to one of three conditions: (a) active contraction of the muscles in the lower part of the face by holding a Chinese chopstick horizontally in the mouth while exerting constant pressure with the teeth; (b) active contraction of the muscles in the upper part of the face by drawing together two small round stickers placed near the inner edge of the eyebrows; or (c) no muscle contraction (control condition). The results showed that the lower manipulation impaired the recognition of happiness and disgust, the upper manipulation impaired the recognition of anger, and both manipulations reduced the recognition of fear.

Interestingly, the studies above using emotional faces as stimuli employed a version of the pen-in-mouth procedure that differed from the original: participants had to hold a pen or chopstick horizontally, not vertically, between their teeth. Furthermore, the authors’ intentions in using this facial manipulation were very different. Blaesi and Wilson (2010) aimed to create a smile-facilitating condition, whereas other studies (Borgomaneri et al., 2020; Oberman et al., 2007; Ponari et al., 2012) aimed to induce irrelevant muscular noise that blocks facial mimicry or interferes with contractions of the lower facial muscles. The lack of consistency in both methodology and intentions across the reported studies may have contributed to the controversial results on the FFH.

### Facial feedback hypothesis and long-term memory

The encoding specificity principle states that it is easier to retrieve a memory when contextual elements present at encoding match those present during retrieval (Tulving & Thomson, 1973). Moreover, research has shown that memory retrieval involves sensorimotor pathways that simulate events that occurred during encoding (for a review, see Ianì, 2019). Thus, understanding how bodily states influence memory is crucial. Dijkstra et al. (2007) investigated the effect of posture congruency at encoding and recall. The authors hypothesized that adopting a posture congruent between encoding and retrieval facilitates recall. To test this, participants were asked to recall autobiographical memories of specific events (e.g., their last dental visit) while taking postures that were either congruent (lying on a recliner with their mouth open) or incongruent (standing upright with their hands on their hips). The results showed that participants recalled past experiences faster when assuming a congruent posture compared to an incongruent posture, both in immediate and delayed recall tests (but see Limata et al., 2021, suggesting that mere congruency between posture at encoding and retrieval does not always enhance memory performance).

Another study by Limata et al. (2023) showed that body posture can influence memory in a recognition task when posture is manipulated in a way that is crucial for the execution or potential execution of actions. During encoding, participants observed a series of objects and performed actions on another series of objects using their upper limbs. During recognition, participants in one group held their hands in front of them (non-interfering posture), while participants in a second group held their hands behind their back (interfering posture). Participants with a non-interfering posture recognized objects on which they had previously performed actions faster than objects they had only observed, but this advantage disappeard for the interfering posture group. These findings suggest that a posture inconsistent with the encoding action can affect memory performance, as reflected in reaction times.

These and other studies (for a review, see Ianì, 2019) provide evidence for a relationship between bodily states and memory. Facial expressions can also be considered bodily states that, when manipulated, may influence mnemonic processes. However, there is still a notable lack of research examining whether facial muscle manipulation using the pen-in-mouth procedure affects memory, particularly when emotional facial expressions are used as stimuli. Only one study has directly addressed this issue. Kuehne et al. (2021) investigated whether manipulating the facial muscles involved in smiling influences the immediate recall of emotional facial expressions. In their study, participants performed a working memory task with happy and sad faces while holding a pen between their teeth (facilitating smiling) or in their non-dominant hand. During encoding, participants were shown happy or sad faces of varying intensities. After each target face, a neutral face appeared, and participants were required to reproduce the target facial expression by scrolling the mouse wheel: upwards to increase the intensity of happiness and downwards to increase the intensity of sadness. The results showed that participants in the teeth condition reproduced happy faces more accurately, suggesting that facilitating smiling selectively enhances immediate memory for happy faces. Notably, this study examined short-term memory for emotional faces, whereas, to our knowledge, no studies have investigated the facial feedback hypothesis in relation to long-term memory for emotional faces.

### Aims of the present study

Based on the reviewed literature, the present study aimed to test the FFH using the original pen-in-mouth procedure in two areas central to theories of embodied emotion: the perception of emotional faces and the long-term memory of emotional faces. More specifically, our aims were fourfold:

- to replicate the effect of the original pen-in-mouth procedure on the perception of positive stimuli using happy facial expressions;
- to examine whether this procedure also influences the perception of neutral and angry facial expressions;
- to clarify inconsistent findings reported in previous research that employed facial expressions as stimuli but used different variants of the pen-in-mouth procedure;
- to investigate whether congruency between the observer’s facial expression and the expression of the observed face enhances long-term memory for the very same emotional faces.

The experiments were approved by the Bioethical Committee of the University of Turin.

## Experiment 1: Do observers’ facial expressions modulate the perception of observed happy, neutral and angry faces?

Experiment 1 focuses on the role that the observer’s facial expression plays in modulating the valence evaluation of happy, neutral, and angry faces. Based on Strack et al. (1988), we hypothesised that when the observer’s muscle activity associated with smiling is facilitated, happy faces should be rated more positively than when it is inhibited. If this prediction holds, the results will provide support for the FFH regarding emotional expression perception. With respect to angry and neutral faces, we do not have specific predictions: to our knowledge, most of the studies on perception employing the pen-in-mouth procedure focused exclusively on positively connoted stimuli. Soussignan (2002) conducted the only study that employed this procedure with participants rating their reaction to both positive and negative emotionally connoted stimuli (i.e., videoclips). The author replicated Strack et al.’s (1988) results with higher positive reactions for positively connoted stimuli when smiling was facilitated. No significant difference was detected in the reactions to negative stimuli, regardless of the position of the pen.

The participants’ task was to rate the emotional valence of happy, neutral, and angry faces on a 7-point Likert scale, ranging from extremely negative to extremely positive. Half of the participants held the pen between their lips, a pose that inhibits smiling, and the other half held the pen between their teeth, a pose that facilitates smiling. We recorded response times for exploratory purposes as well.

## Method

### Participants

To determine the required sample size, an a-priori power analysis based on an effect size of *d* = 0.66 derived from a similar study (Soussignan, 2002) was conducted using G*Power (Version 3.1.9.7; Faul et al., 2007). Since we had a specific hypothesis only for happy faces, we conducted a power analysis for a one-tailed independent-samples *t* test assessing the difference in ratings for happy faces between two groups (teeth vs. lips). The significance level was set at α = 0.05, with desired power (1 – β) = 0.80. The results indicated that a total sample size of 60 participants (30 per group) was required to detect the expected effect.

Participants were 61 students from the University of Turin. Data of one participant were excluded because instead of firmly holding the pen between her lips, she let it slide down and played with it. Therefore, the final sample was composed of 60 participants (26 men, 34 women, mean age = 24.28 years, *SD* = 4.52). Table S1 in the online Supplementary Materials shows the allocation of women and men to the experimental groups (teeth vs. lips). All participants were Italian; a minority of them (*n* = 3) had foreign origins but were grown in Italy and were fluently speaking Italian. Because the FFH is discussed in many psychology classes, we recruited participants from other university courses. They voluntarily participated in the experiment in exchange for academic credit.

### Materials

The material consisted of 18 faces of 18 different actors selected from the KDEF database (Lundqvist et al., 1998), with 6 faces belonging to each of the following categories: happy, neutral, and angry. The faces in each category belong to 3 women and 3 men. We used a 7-point Likert scale ranging from 0 (*extremely negative*) to 6 (*extremely positive*), with intermediate values of 1 (*negative*, 2 (*slightly negative*), 3 (*neutral*), 4 (*slightly positive*), 5 (*positive*), to assess the emotional valence participants assigned to each stimulus. To calculate response times, the Likert scale was structured in a semicircular shape with the mouse automatically positioned equidistantly from each label.

### Procedure

The experiment was conducted in a single, individual session in a quiet room. Participants were randomly assigned to the two experimental groups: in the teeth group they were invited to vertically hold a pen between their teeth (facilitating smile), and in the lips group they were invited to vertically hold a pen between their lips (inhibiting smile) (see Fig. 1 in Strack et al., 1988, p. 771). During the experimental session, participants were video recorded with a hidden mini camera to control whether they held the pen correctly. We made sure that they were not aware of being video recorded to avoid the inhibiting effect on the facial feedback observed in Noah et al. (2018).

At arrival the experimenter asked the participant to sign the informed consent and then to sit in front of a computer to read with the experimenter the following instructions on the computer screen:

Thank you for taking part in this study. You will see a series of photos showing faces. For each photo, you have the task of judging the extent to which the emotion expressed by the person is negative or positive. Shortly after each photo, a scale appears on which you can give your rating from “extremely negative” to “extremely positive”. You must use the mouse to enter your rating. We ask you not to let go of the mouse until the end of the experimental session. The scale is the following:
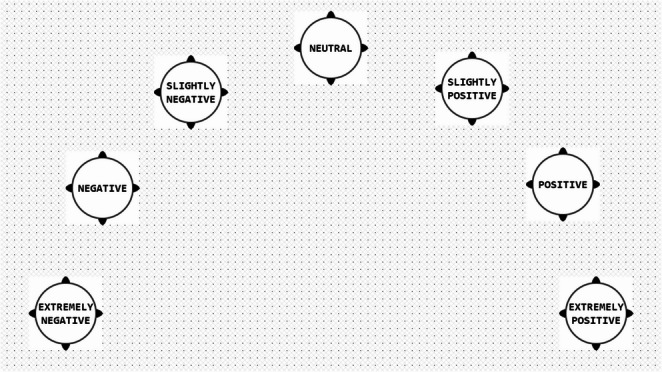

Try to answer spontaneously, without thinking too much about it.

Press the space bar for further instructions.

We also ask you [to hold this pen between your teeth without touching it with your lips/to hold this pen between your lips without touching it with your teeth] for the whole duration of the experimental session. Before you start, we ask you to place the mouse in a position that is comfortable for you. Once the experimental session is finished, we ask you to knock on the door of the room.

Press the space bar when you want to start.

After reading the instructions, right before the beginning of the task, the experimenter made sure that the participant was holding the pen correctly between the teeth or lips and left the room.

Each participant was presented with a total of 18 faces. Each face was displayed for 5 s, after which a response screen with a 7-point Likert appeared. No time limit was imposed for the response; the screen remained visible until the emotional valence rating was provided. After the rating, a new face appeared. Stimuli were presented using E-Prime 3.0 (Psychology Software Tools, 2020). They were shown in random order, with the only constraint that two faces of the same valence were not presented consecutively. Valence ratings and corresponding response times were recorded for each face.

After the experiment, all participants were informed that they had been videotaped (none of them noticed the hidden camera) and asked again whether they consented to the use of their data and the video recording. They then read and signed a post-study consent form and were fully debriefed about the aims of the study.

## Results^1^

**Fig. 1:**
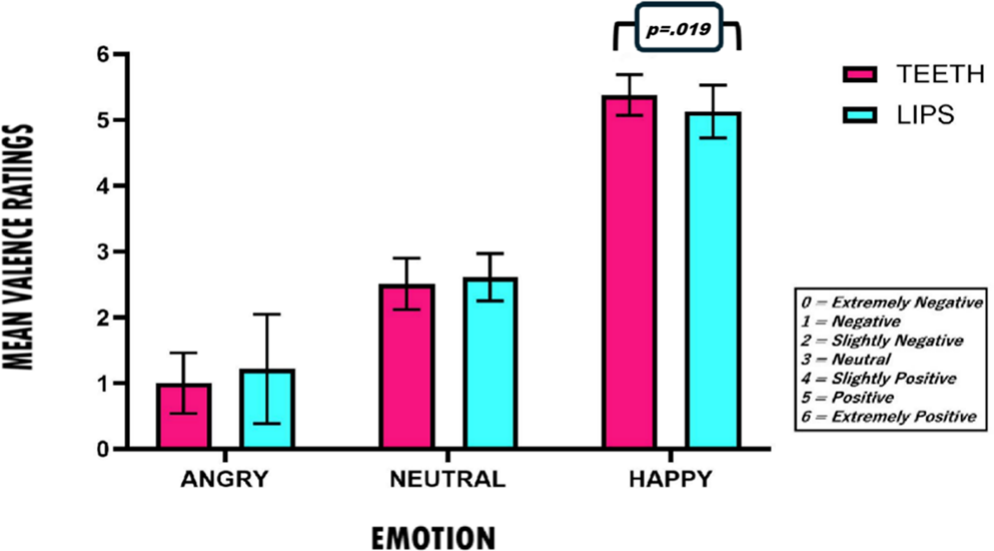
Means and standard deviations for participants’ valence ratings as a function of the type of stimuli and experimental groups (Teeth vs. Lips) in experiment 1

For both experiments, analyses were performed using R (version 4.3.1; R Core Team, 2023). Figure 1 shows a histogram of the participants’ mean ratings for the facial expressions on the emotional valence scale in the two experimental conditions: pen between the teeth and pen between the lips. Means and standard deviations are provided in Table S2 in the online Supplementary Materials.

Normality of valence ratings and response times was assessed using Shapiro-Wilk tests separately for each emotional condition. The results for valence ratings showed significant deviations from normality for neutral (*W* = 0.932, *p* = .002), and angry faces (*W* = 0.878, *p* < .001), whereas happy faces did not show a significant deviation (*W* = 0.961, *p* = .055). For response times, significant deviations from normality were observed across all emotional conditions: happy (*W* = 0.882, *p* < .001), neutral (*W* = 0.872, *p* < .001), and angry faces (*W* = 0.915, *p* < .001). Therefore, nonparametric repeated-measures mixed ANOVAs implemented in the RM() function of the *MANOVA.RM* package in R (Friedrich et al., 2019) were conducted on both, the valence ratings and the response times. The same statistical approach has been previously adopted in memory and perceptual studies where the normality assumption was violated, using the same package and function in the R environment (Borngräber et al., 2022; Hoffmann et al., 2022). Statistical inference was based on ANOVA-type statistics (*ATS*) with parametric bootstrap resampling (5,000 iterations), which provides robust control of Type I error in repeated-measures designs without assuming multivariate normality or sphericity (Bathke et al., 2018).

The 2 (group: teeth, lips) x 3 (emotion: happy, neutral, angry) nonparametric repeated-measures mixed ANOVA on valence ratings showed a significant main effect of emotion (*ATS*(1.69, ∞) = 969.69, *p* < .001), while the main effect of group was not significant (*ATS*(1, 92.34) = 0.09, *p* = .77). Pairwise Wilcoxon signed-rank tests with Bonferroni correction were used to evaluate more in detail the differences in valence ratings across emotional stimuli: angry faces showed lower valence ratings compared to happy, (*z* = -6.74, *p* < .001, *r* = .87) and neutral faces (*z* = -6.45, *p* < .001, *r* = .83), and neutral faces showed lower valence ratings than happy faces (*z* = 6.74, *p* < .001, *r* = .87).

Crucially, the emotion by group interaction was also statistically significant (*ATS*(1.69, ∞) = 3.30, *p* = .048). To follow up this interaction, separate nonparametric repeated-measures ANOVAs were conducted within each group. For the teeth group, the effect of emotion was significant (*ATS*(1.70, ∞) = 956.87, *p* < .001), with means showing highest ratings for happy faces, intermediate ratings for neutral faces, and lowest ratings for angry faces. For the lips group, emotion also had a significant effect on valence ratings, (*ATS*(1.49, ∞) = 300.26, *p* < .001), with means showing the same pattern as in the teeth group. Thus, between-group comparisons were conducted separately for each emotional condition using Wilcoxon rank-sum tests, with Bonferroni correction applied to control for multiple comparisons. A significant group difference emerged only for the happy faces, (*U* = 617, one-tailed *p* = .019, Cliff’s delta = 0.37), with higher valence ratings in the teeth group compared to the lips group. No significant between-group differences were observed for neutral (*U* = 392, two-tailed *p* = 1.00) and angry (*U* = 392, two-tailed *p* = 1.00) faces.

Taken together, these results indicate that the groups differed specifically in their evaluations of happy faces, with the teeth group giving significantly more positive ratings than the lips group, whereas a similar group effect was not observed for neutral and angry faces. This suggests that the classical pen-in-mouth manipulation can affect the emotional processing of happy faces only, whereas this does not apply to neutral and angry faces.

**Fig. 2:**
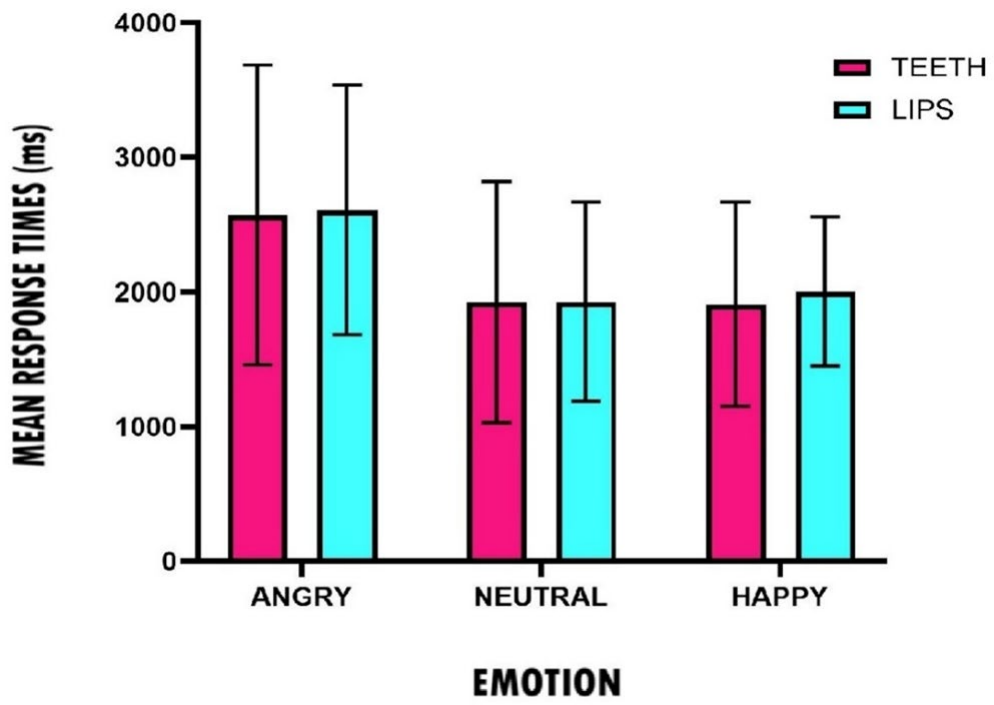
Mean response times (in Milliseconds) and SDs for participants’ ratings as a function of the expressed emotion and experimental groups (Teeth vs. Lips) in experiment 1

Figure 2 shows a histogram of the response times of the participants when evaluating the emotional valence of the faces in the two experimental conditions: pen between the teeth and pen between the lips. Means and standard deviations are provided in Table S3 in the online Supplementary Materials.

A 2 (group: teeth, lips) x 3 (emotion: happy, neutral, angry) nonparametric repeated-measures mixed ANOVA showed a significant main effect of emotion (*ATS*(1.82, ∞) = 22.60, *p* < .001), indicating that participants’ response times differed across the three emotional conditions. Pairwise Wilcoxon signed-rank tests with Bonferroni correction showed that response times for angry faces were significantly higher than for both neutral (*z* = 4.82, *p* < .001, *r* = .62) and happy faces (*z* = 5.37, *p* < .001, *r* = .69). No significant difference in response times was found between happy and neutral faces (*z* = 0.57, *p* = 1.00). No main effect of group was observed (*ATS*(1, 100) = 0.06, *p* = .81), nor was there an interaction effect between emotion and group (*ATS*(1.82, ∞) = 0.09, *p* = .902).

Overall, the results show that, in line with the FFH, happy faces were rated more positively when the activation of the zygomatic muscle was facilitated. This finding is consistent with Strack et al. (1988), where positive stimuli (funny cartoons) were rated more positively in the teeth condition than in the lips condition. Our results also show longer response times when evaluating angry faces compared to happy and neutral faces. This may be because negative stimuli require longer cognitive processing than positive or neutral stimuli due to the threatening values they may carry (Baumeister et al., 2001). However, this result and its interpretation should be treated with caution given the exploratory nature of the response times analyses and the relatively low number of stimuli. 

## **Experiment 2: Do observers’ facial expressions modulate long-term memory of observed happy, neutral and angry faces?**

The aim of Experiment 2 was to investigate whether the observer’s facial expressions modulate the memory for previously seen emotional faces. Using the same facial manipulation as in Experiment 1, we tested whether congruency between the observer’s facial expression and the emotion conveyed by the observed face favors recognition.

The experiment consisted of two phases. In the encoding phase, participants observed 24 stimuli, equally divided into 8 happy, 8 neutral, and 8 angry faces. To ensure they processed the emotional expression, participants were asked to rate the positive or negative emotional state of the person depicted in each photo. In the recognition phase, participants were presented with 24 screens, each depicting the same actor with a happy, neutral, and angry face. For each actor, only one of the three facial expressions had been shown during encoding. Participant’s task was to identify this expression while going through the pen-in-mouth procedure. The rationale behind this procedure was as follows: although a straightforward approach would have been to use different actors as filler faces during recognition, such a design cannot rule out the possibility that idiosyncratic facial features (e.g., freckles, moles) influence memory performance. To avoid this potential confound, during recognition, we presented the same actors as in encoding, with filler faces differing from targets only in the emotion expressed (happy, neutral, angry).

## Method

### Participants

A power analysis revealed that at least 24 participants were required to reach an appropriate statistical power of 0.95 to detect a significant effect (with α = 0.05), assuming a medium effect size (*f* = 0.35, η_p_^2^ = 0.109), the same found by Kuehne et al. (2021), who conducted a similar study on the effect of facial expression manipulations on immediate recall. The sample size was determined using the G-Power software (Version 3.1.9.6; Faul et al., 2007). To recruit participants, we offered students from the University of Turin the opportunity to earn credits. The participants had not taken part in Experiment 1. All participants were Italian. For the same reasons discussed in Experiment 1, we recruited students not attending a psychology course.

We tested a total of 28 participants but data from 2 participants was removed from the analyses: one participant was aware of the FFH and the pen-in-mouth procedure, while a second participant encountered a technical problem (blocking of the response keys) during the experiment. Thus, the final sample consisted of 26 participants (14 men, 12 women, mean age = 24.41 years, *SD* = 4.31). Table S1 in the online Supplementary Materials shows the allocation of women and men to the experimental groups (teeth vs. lips).

### Materials

The material consisted of 72 pictures of 24 different actors from the KDEF database (Lundqvist et al., 1998). During encoding, 24 stimuli were presented, including 8 actors with a happy expression, 8 with a neutral expression, and 8 with an angry expression. During retrieval, the same 24 actors were presented again, each with three different facial expressions - happy, neutral, and angry - yielding a total of 72 pictures.

### Procedure

The experiment was conducted in a single, individual session in a quiet room. As in Experiment 1, participants were randomly assigned to the lips or the teeth condition.

For both groups, the experiment consisted of two phases, encoding and recognition, interrupted by a distractor task. At their arrival participants were asked to read and sign the informed consent form. Then they were asked to sit in front of a computer to begin the encoding phase. The following instructions were read by the participant together with the experimenter:

Thank you for your participation. You will be presented with photos of faces and your task is to rate how negative or positive the emotion expressed by the person is. Immediately after each photo is presented, a scale appears that allows you to rate it from extremely negative to extremely positive. Please use the mouse to express your evaluation.

Try to answer spontaneously without thinking too much about it.

Press the space bar when you want to start.

During the encoding phase, each participant was presented with 24 faces for 5 s each. Immediately after the presentation of each face, the same 7-point Likert scale as used in Experiment 1 appeared on the screen to let participants rate the emotional valence of the face they had just seen. The rating task had the goal to ensure that the participants’ attentive resources were directed towards the stimuli and the ratings obtained were not considered as a dependent variable.

After completing the encoding phase, each participant performed a ten-minute distractor task consisting in finding differences between pairs of vignettes. No faces or emotions were shown in these cartoons to avoid interference with the experimental material. Then the experimenter entered the room and read together with the participant the following instructions for the recognition phase:

In this phase you will see a series of screens, each composed of three photos of the same actor. In each screen, one of the three photos has already been presented to you in the previous phase: your task is to identify it.

To answer, press the left arrow to indicate the photo shown on the left, the down arrow to indicate the central image, the right arrow to indicate the photo shown on the right of the screen.

We ask you to give the answer when you are sure by pressing the corresponding key.

For the duration of the experiment, keep the fingers of one hand resting on the labeled keys.

We also ask you [to hold this pen between your teeth/lips without touching it with your lips/teeth].

Press the space bar when you want to start.

**Fig. 3:**
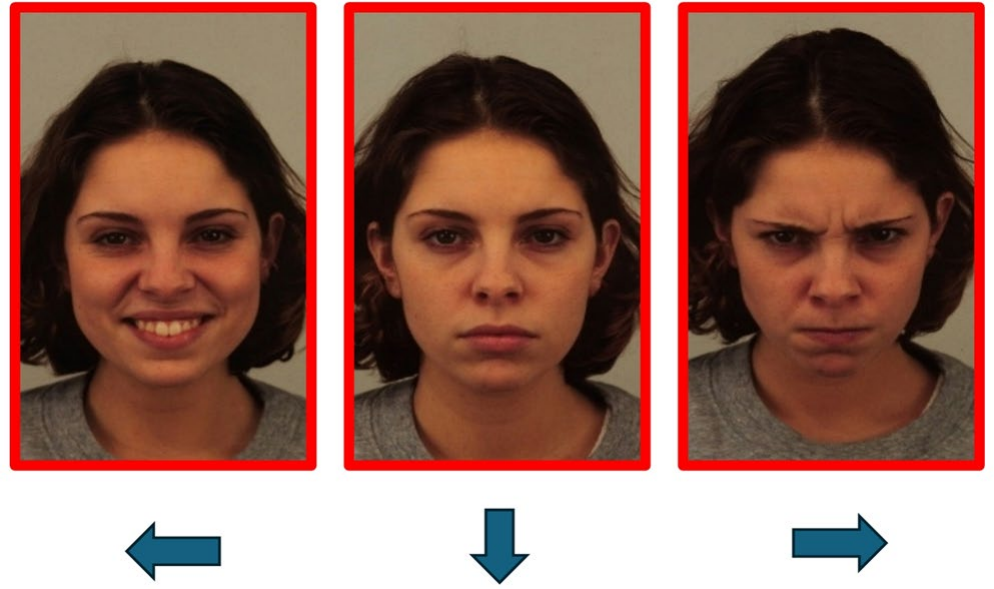
Example of a screen shown to the participants in experiment 2 during recognition. Source: KDEF (Lundqvist et al., 1998). Identification codes of stimuli: left (happy): AF20HAS, middle (neutral): AF20NES, right (angry): AF20ANS

After reading the instructions and before leaving the room, the experimenter made sure that the participant was holding the pen correctly between the teeth or lips. During this phase, participants in both groups were presented with 24 screens, each displaying three photographs of the same actor: one with an angry expression, one with a neutral expression, and one with a happy expression (see Fig. 3).

For each displayed screen, only one of the three photographs was shown during encoding. The participant’s task was to identify the previously seen photo by pressing one of the three QWERTY-keyboard keys V, B, N, respectively labeled with a left, down, or right arrow. To permit an accurate measurement of response times, participants were instructed to keep three fingers of the same hand resting on the response keys throughout the whole recognition phase. Stimuli were presented using E-Prime 3.0 (Psychology Software Tools, 2020). We recorded the recognition accuracy and the response times for each displayed stimulus. The order of presentation of the different actors was randomized as well as the position of the different emotional expressions. In this phase, the participants were video recorded with a hidden mini camera. The purpose of the video recording was to ensure that the pen was well positioned, and that the participant kept three fingers of the same hand resting on the answer keys for the entire duration of the task.

After completing the recognition task, the experimenter asked the participant to read and sign the post-study consent form where they were informed that they had been videotaped. After obtaining consent for this use, the experimenter shared with each participant the aims of the study and asked if they had noticed that they had been video recorded (none of them noticed the hidden camera) and if they had any previous knowledge about the FFH or the pen-in-mouth procedure.

## Results

**Fig. 4:**
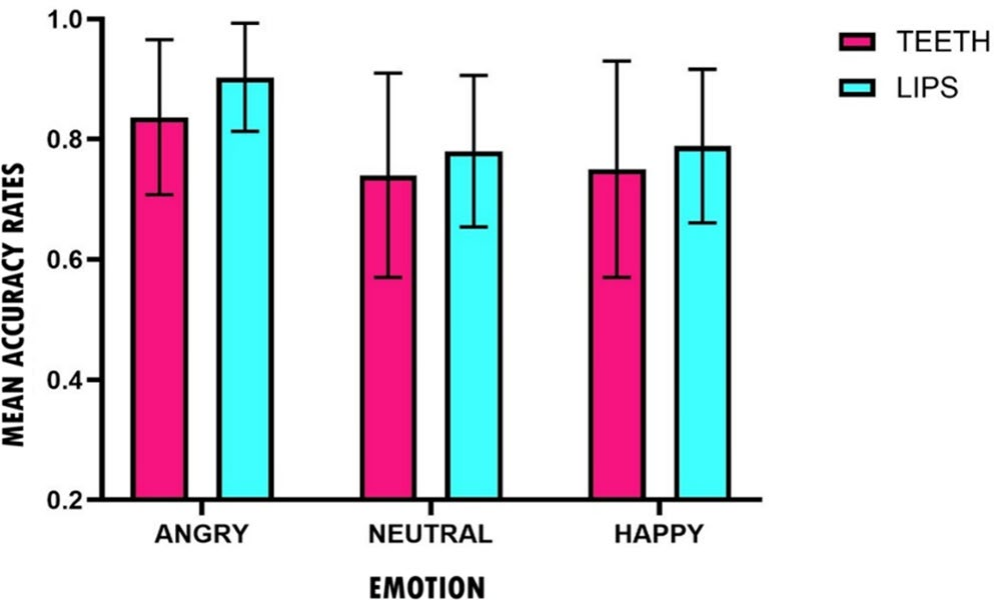
Accuracy rates (Ms and SDs) for face recognition as a function of the type of stimuli and the group (Teeth vs. Lips) in experiment 2

Figure 4 shows a histogram of the participants’ mean accuracy rates in discriminating seen and unseen faces as a function of the displayed emotion (angry, neutral, happy) and group (teeth vs. lips). Means and standard deviations are provided in Table S4 in the online Supplementary Materials.

Normality of accuracy rates and response times was assessed using Shapiro–Wilk tests separately for each emotional condition. The results for accuracy ratings revealed significant deviations from normality for angry (*W* = 0.853, *p* = .002), happy (*W* = 0.908, *p* = .024) and neutral faces (*W* = 0.787, *p* < .001), while the results for response times indicated a significant deviation for neutral faces (*W* = 0.899, *p* = .015). Therefore, as in Experiment 1, we conducted nonparametric repeated-measures mixed ANOVAs implemented in the RM() function of the *MANOVA.RM* package in R (Friedrich et al., 2019) on accuracy ratings as well as response times. Statistical inference was based on *AST* with parametric bootstrap resampling (5,000 iterations).

The 2 (group: teeth, lips) x 3 (emotion: angry, happy, neutral) nonparametric repeated-measures mixed ANOVA analysis conducted on accuracy ratings revealed a main effect of emotion (*ATS*(1.91) = 6.72, *p* = .04). Pairwise Wilcoxon signed-rank tests with Bonferroni correction showed that accuracy for angry faces was significantly higher than for both happy (*z* = 2.73, *p* = .020, *r* = .53) and neutral faces (*z* = 3.02, *p* = .008, *r* = .59). There was no significant difference in accuracy between the happy and neutral faces (*z* = -0.13 *p* = 1.00).

No main effect of group was found (*ATS*(1) = 1.49, *p* = .237), neither an interaction effect between emotion and group (*ATS*(1.91) = 0.12, *p* = .881).

**Fig. 5:**
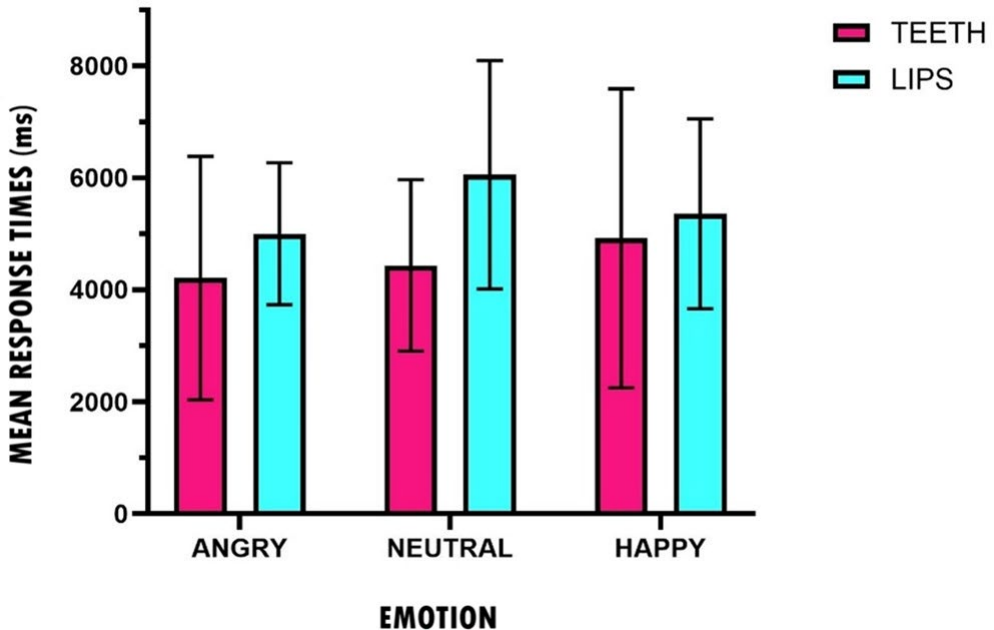
Mean response times in milliseconds and SDs of face recognition for target stimuli as a function of the emotion they expressed and of the group (Teeth vs. Lips) in experiment 2

Figure 5 shows a histogram of the mean response times for discriminating seen and unseen faces as a function of the emotion expressed and the group (teeth vs. lips). Means and standard deviations are provided in Table S5 in the online Supplementary Materials.

The 2 (group: teeth, lips) x 3 (emotion: angry, happy, neutral) nonparametric repeated-measures mixed ANOVA analysis conducted on response times revealed no significant main effect of emotion (*ATS*(1.73, ∞) = 1.62, *p* = .201), no significant main effect of group, (*ATS*(1, 36.87) = 2.29, *p* = .138), nor an interaction effect between emotion and group (*ATS*(1.73, ∞) = 1.26, *p* = .281).

Overall, there was no effect of facial feedback on long-term memory for emotional faces. Furthermore, the results show that angry faces were better recognized in the long-term memory task than neutral and happy faces. This advantage for angry facial expressions may be explained by their greater evolutionary relevance: threatening stimuli tend to capture attention more strongly (as shown by the longer response times for angry faces in Experiment 1) and to be encoded more deeply in memory. This is consistent with Kensinger (2007), who showed that the valence of a memory influences recall accuracy, with negative memories remembered in greater detail than positive ones. Similarly, our results suggest that negative emotional stimuli, such as angry faces, may enhance long-term memory retention due to their intrinsic evolutionary significance as a sign of potential threat.

## General discussion

The present study examined the role of facial feedback in the perception and memory of emotional faces by employing the pen-in-mouth procedure across two experiments, using different emotional facial expressions (happy, neutral, and angry) as stimuli.

Experiment 1 demonstrated that inducing participants to hold a smile-facilitating posture (pen between teeth) affected their emotional perception, leading them to rate happy faces more positively than those with a smile-inhibiting posture (pen between lips). There was no significant difference between the teeth and lips conditions for neutral and angry faces. These results are consistent with Strack et al. (1988), who observed - using the pen-in-mouth procedure - that funny cartoons were rated more positively in the smile-facilitating condition than in the smile-inhibiting condition. Furthermore, our results suggest that the same facial manipulation cannot affect the processing of neutral and angry faces. This finding may be of interest to the broader debate on epistemological assumptions in cognitive science in general, and specifically in the embodied cognition literature (Ianì, 2024). Our data indicate that the effects of facial manipulation depend on the specific materials and procedures used in the experiment.

In this regard, it is not easy to compare our findings with studies that have used different facial manipulations to investigate the FFH. For example, Coles et al. (2019) conducted a meta-analysis and, by considering 286 effect sizes from 138 studies, found a significant, though small, overall effect of facial feedback on subjective emotional experience and/or affective judgements. However, the studies included in this meta-analysis also used facial manipulations that differ from the pen-in-mouth procedure, such as facial mimicry and the directed facial action task.

Given the contrast between the failed replication study by Wagenmakers et al. (2016) reported in the introduction and the results of Coles et al. (2019) providing evidence in favor of the FFH, Coles et al. (2022) conducted a further multi-lab test involving 26 laboratories and 3,878 participants from 19 countries. Participants rated either positive or neutral pictures while holding either a neutral or a happy facial expression. To elicit a happy expression, participants completed the task in one of the following three conditions: (a) mimicry of images of actors displaying happiness, (b) voluntary facial action task, or (c) pen-in-mouth procedure. The authors found a clear facial feedback effect when facial mimicry and the voluntary facial action task were used, while the pen-in-mouth procedure produced less conclusive evidence. Nonetheless, when some participants’ inclusion criteria were relaxed (e.g. performing the task on a device other than a laptop or personal computer), the analysis provided strong support for the facial feedback effect in the pen-in-mouth task as well. Notably, participants were significantly less aware of the study purposes in the pen-in-mouth condition compared to the two other facial manipulation conditions. Our results from Experiment 1 provide further evidence that, by using the original pen-in-mouth procedure - which appears to be less susceptible to demand characteristics - the FFH is supported when happy faces are used as stimuli.

Experiment 2 revealed no significant effect of participants’ facial expressions on recognition accuracy for previously seen emotional faces, suggesting that the influence of facial feedback may be limited to the perceptual domain rather than memory processes. This finding does not align with previous studies showing that postural congruency between encoding and recall or recognition can influence memory accuracy (Dijkstra et al., 2007; Limata et al., 2023), and that facial feedback may affect the immediate recall of previously seen emotional faces (Kuehne et al., 2021). This discrepancy could be due to methodological differences between our study and previous ones. Specifically, the congruency effect we expected has mainly been examined by considering the match between body posture at encoding and retrieval (Dijkstra et al., 2007), whereas we considered the congruency between the participant’s facial expression and the observed one, and did so only during the retrieval phase. Furthermore, we tested the congruency effect on long-term memory, rather than on immediate recall for emotional faces, as in Kuehne et al. (2021). To our knowledge, no previous study has directly examined the relationship between facial expression congruency and long-term memory. Moreover, the results of the study by Limata et al. (2023), summarized in the introduction, indicate that body posture can influence memory in a recognition task when posture is manipulated in a way that is crucial for the execution or potential execution of actions. This might lead us to expect that, in Experiment 2, the teeth condition would have facilitated the production of a smile and, in turn, the recognition of positive stimuli during retrieval; however, this was not the case. A possible explanation for these results is that our stimuli were too obvious. Wood et al. (2016) suggest that “sensorimotor simulation may be especially useful for emotion recognition when the perceived expression is subtle or ambiguous” (p. 8). An alternative explanation for the absence of a facilitating effect may be that we tested the congruency between expression and memory only during the retrieval phase, which may have underestimated the potential facilitatory effect of facial manipulation on memory. If we had also manipulated it during encoding, memory performance might have been enhanced through dual coding of the same stimulus (Paivio, 1986), providing multiple retrieval cues that could have increased the likelihood of remembering positive stimuli. Similarly, research on embodied mental imagery has shown that encoding information through both sensory and motor simulations can strengthen memory traces (Marre et al., 2021). In Limata et al.’s study (2023), participants may have formed both a memory trace linked to body movement and a trace linked to the visual perception of the object.

Experiment 2 showed that, regardless of facial manipulation, angry faces are recognized more accurately than neutral and happy faces, and Experiment 1 suggests that angry faces require more attentional resources to be evaluated. Although these results should be interpreted with caution, particularly regarding response times in Experiment 1, they are consistent with studies indicating that our cognitive resources are biased towards enhanced recognition and deeper processing of potentially harmful stimuli (Barros et al., 2023). Our findings support the idea that threatening stimuli are intrinsically more relevant due to their importance for survival, reflecting the well-documented negativity bias in information processing (Baumeister et al., 2001; Kensinger, 2007), which has been shown to enhance the processing and memory of negative emotional stimuli. This may be especially true for emotional faces, which are highly relevant for adaptation in daily life (Wood et al., 2016).

Overall, the results of the two experiments indicate that manipulating facial muscles using the original pen-in-mouth procedure influences perception, but not long-term memory for emotional faces.

### Limitations

Since our a-priori power analysis to determine the sample size for Experiment 1 was based on a directional hypothesis regarding valence ratings for happy faces, results concerning neutral and angry faces should be interpreted with caution, particularly with respect to the response times observed for angry faces. Similarly, the sample size in Experiment 2 (*N* = 26) is relatively small, which may affect the robustness of the findings.

Another possible limitation of our study is that the results may have been influenced by differences in the difficulty of holding the pen in the lips versus the teeth condition. We did not assess this variable, as Strack et al. (1988) found no differences in difficulty between the two pen-in-the-mouth conditions.

Finally, the generalizability of findings in emotional research is often limited by cultural differences. Mesquita and Frijda (1992) highlight that emotional evaluations are shaped by culture, suggesting that results from one cultural group may not be universally applicable. Our study, conducted exclusively with W.E.I.R.D. (Western, Educated, Industrialized, Rich, and Democratic) students, reflects this limitation. Future research should consider cultural and socio-economic variability to ensure broader applicability of findings.

## Conclusions and future research

Our findings contribute to a deeper understanding of the FFH by delineating its boundary conditions across different domains, particularly perception and long-term memory for emotional faces. Taken together, the results of our two experiments indicate that facial muscle activity plays an important role in modulating the perception of emotional expressions. However, the influence of facial feedback on long-term memory is not clear, suggesting that perceptual and memory processes may rely on facial muscle feedback in different ways. This underscores the need for further investigation into the effects of facial feedback across different cognitive domains and highlights the complexity of embodied emotion theories (Barsalou, 2008; Price et al., 2015; Prinz, 2004). Future research could investigate the FFH by not only manipulating the expression of happiness using the pen-in-the-mouth procedure, but also manipulating the expression of anger (as done, for example, by Ponari et al., 2012) and assessing its effects on the perception of emotional faces. Furthermore, regarding the possible effect of facial feedback on memory, future studies could examine whether facial manipulations during both encoding and retrieval have a potential facilitatory effect on memory.

## Practical implications

The relevance of the present study lies in its potential impact on social interaction. Several studies have shown that facial mimicry can enhance affiliation, prosocial behavior, and empathy (e.g., Hess, 2021; Lakin & Chartrand, 2003). This is particularly relevant in psychotherapeutic settings. In psychotherapy, facial expressions are a central channel of communication between patients and therapists (Bänninger-Huber, 1992). The results of our study suggest that therapists should be aware that their own emotional states may influence how they perceive patients’ emotions. For example, being in a positive emotional state may lead therapists to perceive patients as happier than they are. Conversely, patients’ facial expressions may influence therapists’ emotional states through subtle processes of facial mimicry and sensorimotor simulation (Wood et al., 2016). These processes are consistent with the broader phenomenon of emotional contagion, whereby individuals tend to automatically mimic and internalize the emotional expressions of others (Hatfield et al., 1994). This dynamic may create a feedback loop: for example, an angry facial expression displayed by a patient may elicit corresponding feelings in the therapist, which could in turn be expressed through subtle facial cues. These cues may then reinforce the patient’s own emotional state, potentially intensifying the emotional exchange during the interaction.

## Supplementary Information

Below is the link to the electronic supplementary material.

Supplementary Material 1

## Data Availability

The data reported in this paper are archived at the following link: https://osf.io/ruvhm/overview? view_only=f08427dbfa1a44e98496b2ab69b2fd6b.

## Footnotes

1 Preliminary results of Experiment 1 involving 26 out of 60 participants have been accepted for publication as research notes in the Giornale Italiano di Psicologia (Ras et al., in press)
