# Supplementary material for: Facial feedback on the perception and memory for emotional faces
Source: Psychol Res. 2026 May 11;90(3):89. doi: 10.1007/s00426-026-02307-4 (PMC13161003; doi:10.1007/s00426-026-02307-4)
Supplement: Supplementary file 1 — Supplementary Material 1 [file 426_2026_2307_MOESM1_ESM.docx]

**Supplementary materials**

**Table S1**

*Allocation of Men and Women to the Groups (Teeth vs Lips) in the two Experiments*

|  |  | Teeth | |  | Lips | |
| --- | --- | --- | --- | --- | --- | --- |
| Experiment |  | Men | Women |  | Men | Women |
| Exp 1 |  | 13 | 17 |  | 13 | 17 |
| Exp 2 |  | 6 | 7 |  | 8 | 5 |

**Table S2**

*Mean Ratings (SDs in Parentheses) of Faces on the Emotional Valence Scale (Values from 0 to 6) as a Function of the Emotion They Expressed and the Experimental Group (Teeth vs Lips) in Experiment 1*

|  |  | Group | | |
| --- | --- | --- | --- | --- |
| Stimuli |  | Teeth |  | Lips |
| Angry |  | 1.00 (0.46) |  | 1.22 (0.83) |
| Neutral |  | 2.51 (0.39) |  | 2.61 (0.36) |
| Happy |  | 5.38 (0.31) |  | 5.13 (0.40) |

**Table S3**

*Mean Response Times in Milliseconds (SDs in Parentheses) When Participants Rated the Faces on the Emotional Valence Scale as a Function of the Emotion They Expressed and the Experimental Group (Teeth vs Lips) in Experiment 1*

|  |  | Group | | |
| --- | --- | --- | --- | --- |
| Stimuli |  | Teeth |  | Lips |
| Angry |  | 2573 (1113) |  | 2611 (929) |
| Neutral |  | 1927 (896) |  | 1927 (740) |
| Happy |  | 1909 (759) |  | 2005 (554) |

**Table S4**

*Mean Accuracy Rates (SDs in parentheses) of Face Recognition as a Function of the Emotion They Expressed and of the Group (Teeth vs Lips) in Experiment 2*

|  |  | Group | | |
| --- | --- | --- | --- | --- |
| Stimuli |  | Teeth |  | Lips |
| Angry |  | 0.84 (0.13) |  | 0.90 (0.09) |
| Neutral |  | 0.74 (0.17) |  | 0.78 (0.13) |
| Happy |  | 0.75 (0.18) |  | 0.79 (0.13) |

**Table S5**

*Mean Response Times in Milliseconds (SDs in Parentheses) of Facial Expression Recognition as a Function of the Emotion Expressed and of the Group (Teeth vs Lips) in Experiment 2*

|  |  | Group | | |
| --- | --- | --- | --- | --- |
| Stimuli |  | Teeth |  | Lips |
| Angry |  | 1679 (657) |  | 1627 (813) |
| Neutral |  | 1884 (858) |  | 1583 (824) |
| Happy |  | 1868 (1103) |  | 1413 (730) |
